# Skeletal and Cardiac Muscle Disorders Caused by Mutations in Genes Encoding Intermediate Filament Proteins

**DOI:** 10.3390/ijms22084256

**Published:** 2021-04-20

**Authors:** Lorenzo Maggi, Manolis Mavroidis, Stelios Psarras, Yassemi Capetanaki, Giovanna Lattanzi

**Affiliations:** 1 Neuroimmunology and Neuromuscular Diseases Unit, Fondazione IRCCS Istituto Neurologico Carlo Besta, 20133 Milan, Italy; 2 Center of Basic Research, Biomedical Research Foundation, Academy of Athens, 11527 Athens, Greece; emavroeid@bioacademy.gr (M.M.); spsarras@bioacademy.gr (S.P.); ycapetanaki@bioacademy.gr (Y.C.); 3 CNR Institute of Molecular Genetics “Luigi Luca Cavalli-Sforza”, Unit of Bologna, 40136 Bologna, Italy; 4 IRCCS Istituto Ortopedico Rizzoli, 40136 Bologna, Italy

**Keywords:** desmin, lamin A/C, synemin, desminopathy, muscular laminopathies, cardiomyopathy, secretome, mechanosignaling, nuclear positioning

## Abstract

Intermediate filaments are major components of the cytoskeleton. Desmin and synemin, cytoplasmic intermediate filament proteins and A-type lamins, nuclear intermediate filament proteins, play key roles in skeletal and cardiac muscle. Desmin, encoded by the *DES* gene (OMIM *125660) and A-type lamins by the *LMNA* gene (OMIM *150330), have been involved in striated muscle disorders. Diseases include desmin-related myopathy and cardiomyopathy (desminopathy), which can be manifested with dilated, restrictive, hypertrophic, arrhythmogenic, or even left ventricular non-compaction cardiomyopathy, Emery–Dreifuss Muscular Dystrophy (EDMD2 and EDMD3, due to *LMNA* mutations), *LMNA*-related congenital Muscular Dystrophy (L-CMD) and *LMNA*-linked dilated cardiomyopathy with conduction system defects (CMD1A). Recently, mutations in synemin (*SYNM* gene, OMIM *606087) have been linked to cardiomyopathy. This review will summarize clinical and molecular aspects of desmin-, lamin- and synemin-related striated muscle disorders with focus on *LMNA* and *DES*-associated clinical entities and will suggest pathogenetic hypotheses based on the interplay of desmin and lamin A/C. In healthy muscle, such interplay is responsible for the involvement of this network in mechanosignaling, nuclear positioning and mitochondrial homeostasis, while in disease it is disturbed, leading to myocyte death and activation of inflammation and the associated secretome alterations.

## 1. Intermediate Filament Proteins

The cytoskeleton is formed by three different protein networks: microfilaments, constituted by actin, microtubules, which are polymers of tubulins, and intermediate filaments, which are formed by intermediate filament proteins (IFs). IFs are encoded by over 70 genes differentially expressed in a cell and tissue-specific manner [1]. IFs expression is also influenced by the developmental stage of cells and tissues and by a plethora of disease states [1,2]. Based on similarities in the amino acid sequence and molecular structure, IFs are divided into six subgroups, named type I to type VI [3]. In particular, acidic keratins are type I IFs, basic keratins are type II IFs, desmin, GFAP, perypherin, vimentin and syncoilin are type III IFs, nestin, neurofilament, synemins and internexin are type IV IFs, lamins are type V IFs and lens-specific phakinin and filensin are type VI IFs [3,4]. All IFs share a common structural organization including a central alpha-helical “rod domain” flanked by non-alpha-helical N-terminal (head) and C-terminal (tail) domains. The central alpha-helical domain is formed by three segments separated by two linkers: coil 1A; linker L1; coil 1B; linker L12; and coil 2 [3,4]. This structure assembles into higher order parallel filaments of about 10 nm that contribute to resilience and flexibility in cells subjected to mechanical forces [1,5]. The amino-terminal domains of IFs are necessary for protein assembly, whereas the carboxy-terminal domains are involved in organization of the IFs network and functional protein interactions, mostly regulated by post-translational modifications [4,5]. IFs are located in the cytoplasm, as keratins, desmin, vimentin, GFAP, neurofilaments, internexin, synemin, peripherin and nestin that are components of the cytoskeleton, or in the nucleus as lamins, where they form the nucleoskeleton [3,6]. Lamins, which are distinguished into A and B type lamins, share structural features of cytoplasmic IFs, but form thinner filaments of about 3.5 nm and hold a nuclear localization signal [1,7,8].

## 2. Striated Muscle Intermediate Filament Proteins

### 2.1. Expression of Muscle Intermediate Filament Proteins

IFs can be ubiquitous or tissue-specific proteins and many of them can be considered differentiation markers of a given cell type [9,10]. Several IFs are expressed in striated muscle, some of them being already present in mesenchymal stem cells and myogenic precursors, while others appearing upon cellular determination [10]. Nestin, a type VI intermediate filament, is found at the very early stages of stem cell differentiation and it is maintained in adult skeletal myoblasts, but not in cardiomyocytes [3]. In proliferating myoblasts, vimentin, type III intermediate filament, is widely expressed. However, in differentiated cardiomyocytes, vimentin is replaced by tissue-specific IFs [3]. Synemin is a type IV intermediate filament expressed in a high number of cells, including smooth and striated muscle cells [11,12]. Synemin is also considered a focal adhesion protein due to its localization at the cell membranes, including muscle costameres, where synemin interacts with desmin [3]. Moreover, synemin has been recently implicated in the response to DNA damage as a modulator of non-homologous end-joining in a complex including DNA-protein kinase catalytic subunit [13].

**Figure 1:**
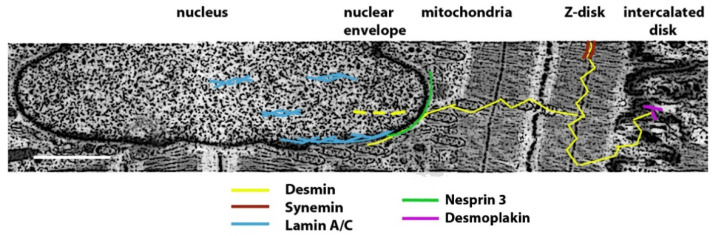
Schematic representation of intermediate filament proteins desmin, lamin A/C and synemin distribution in myocardium. Desmin connects the Z-disks with the intercalated disks, mitochondria, lysosomes (not shown here) and the nuclear periphery. Synemin binds desmin at costameres (not shown here), the membrane units at the Z-disks. Desmoplakin connects desmin to plakoglobin at the intercalated disks. Nesprin 3 binds desmin at the nuclear periphery of cardiomyocytes. Lamin A/C is localized at the nuclear lamina, beneath the inner nuclear membrane and connects to nesprin 3 in the LINC complex (see text). Lamin A/C is also localized in the nucleoplasm. Desmin can transiently enter the nucleus (dotted yellow lines) in cardiac stem cells and interact with transcription factors [16]. In skeletal muscle, similar interactions are established at the Z-disks, mitochondria, lysosomes and costameres between desmin, lamin A/C or synemin and their binding partners [14]. Scale bar, approx. 1 μm.

Desmin is a tissue-specific type III intermediate filament expressed in skeletal and cardiac myocytes, as well as smooth muscle cells [6]. In skeletal and cardiac muscle, desmin surrounds the Z- discs and links the myofibrillar structure to the sarcolemma, the nucleus, mitochondria and lysosomes [6,14,15], while, specifically in cardiac muscle, desmin also connects the Z- discs with the intercalated discs [3] (Figure 1).

In skeletal muscle, desmin binds other IFs as synemin, paranemin, syncoilin, nestin, vimentin, and it is able to bind keratins K8 and K19 [14]. Moreover, binding to the linker protein plectin 1 allows attachment of desmin to the myocyte nucleus [17] through plectin- nesprin interaction [18]. In cardiomyocytes, desmin binds synemin at the costameres, the membrane units surrounding the Z-disks [3,11]. Moreover, desmin connects to the desmosomes through plectin 1f [17,19] and desmoplakin, which in turn binds plakoglobin, a desmosomal armadillo protein [20,21]. Desmoplakin can connect desmin to the desmosomal protein desmoglein either directly or indirectly through plakoglobin. In the heart, this complex protein interplay maintains structural connections during cardiomyocyte contraction [21] and contributes to desmin attachment to the intercalated disks, the gap junction channels, mostly formed by connexin 43, that allow the electrical coupling between neighboring cardiomyocytes [22].

Lamins are type V IFs almost ubiquitously expressed in differentiated cells. However, A type lamins play a major role in skeletal and cardiac myocytes, epithelia and adipose tissue, while B type lamins play a relevant role in the central nervous system [23]. A type lamins, mainly represented by lamins A and C, major splicing products of the *LMNA* gene, form the nucleoskeleton beneath the inner nuclear membrane and functionally interact with nuclear envelope proteins and chromatin-associated factors. Lamins can also directly bind chromatin and regulate specific regions called lamina-associated domains or LADs or indirectly regulate gene transcription through transcription factor binding at the nuclear periphery or acting as platforms for transcriptional regulators [24]. Moreover, lamins play a major role in mechanosignaling through their interaction with the LINC complex (linker of nucleoskeleton and cytoskeleton), including SUN1, SUN2 and the nesprins [23,25,26].

### 2.2. Structure of Muscle Intermediate Filaments

**Figure 2:**
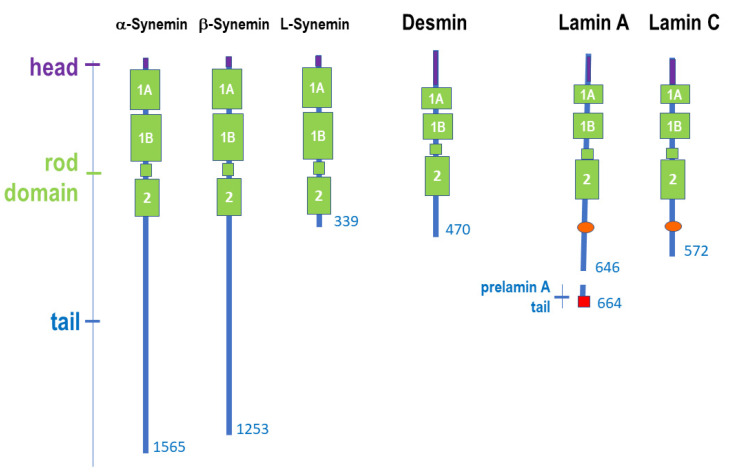
Schematic representation of α-Synemin, β-Synemin, L-Synemin, Desmin, Lamin A and Lamin C protein structure. Each intermediate filament protein has: a head domain (purple); a rod domain including coil1A (1A), coil 1B (1B), a linker domain called L12 (small green box) and coil 2 (2); a tail domain (blue), which can be of variable length even in protein isoforms. Lamin A and C have a globular domain (orange) in their tail. Prelamin A-specific tail with C-terminal CAAX box (red) is also represented. Prelamin A tail is removed upon protein post-translational processing to yield mature Lamin A. α-Synemin, β-, Synemin and L-Synemin are splicing products of *SYNM* gene, Desmin is the product of *DES* gene, Lamin A and C are splicing products of *LMNA* gene. The number of amino acids is indicated at the C-terminus for each protein.

The three synemin isoforms, alpha-, beta- and L-synemin, are produced by the *SYNM* gene (OMIM *606087) on chromosome 15q26.3, through alternative splicing. Desmin is produced by the *DES* gene (OMIM *125660) located on chromosome 2q35, while lamin A and lamin C are splicing products of the *LMNA* gene (OMIM *150330) on chromosome 1q22. Synemin, desmin, lamin A and lamin C share the typical protein structure of intermediate filament proteins. A schematic representation of the protein structure is shown in Figure 2. The head domain is of different length in the three proteins. Synemin has a short head domain of only 10 amino acids, desmin a 112 amino acid head domain, lamins share a head domain of 28 amino acids. Although so far considered an unstructured domain, recent studies have shown that the desmin head domain can transiently be more structured in a way that facilitates protein binding, as in the case of PKA recruitment aimed at desmin phosphorylation [27]. The IFs rod domain is formed by three alpha-helical coiled coil regions (coil 1A, coil 1B and coil 2) and two linker segments (called L1 and L12) in all three types of intermediate filament proteins. The unstructured tail domains are of variable length even within protein isoforms (Figure 2). Alpha- and beta-synemin are much longer than the other intermediate filament proteins, but L-synemin is shorter [11]. Lamin A and C have an IgG-like globular domain in their tails. Lamin A is produced as a longer protein precursor known as prelamin A, which is cleaved upon post-translational processing of its C-terminal CAAX box [23,25,28,29].

Synemin assembles into heteropolymers formed with other IFs in a developmental and tissue-specific fashion [11]. This peculiarity is also observed during muscle development as synemin co-polymerizes with vimentin in myoblasts and desmin in mature muscle [11]. Lamin A and lamin C co-polymerize (so that they are often referred to as lamin A/C) and assemble laterally to form dimers and tetramers [7]. The lamin tetramers further assembles longitudinally into high-order filamentous structures through overlap of coil 1A with coil 2 C-terminal region, in a mode called eA22, which is affected by *LMNA* pathogenetic mutations [30]. However, other assembly modes have been also suggested, as summarized in a recent review article [4]. As mentioned above, the lamin A filaments have a diameter of 3.5 nm, differently from all other IFs [7], and lamin A coil 1B has a specific 42 residues insert required for dimer and tetramer formation [7], a globular domain in the tail and a lamin A-specific C-terminal CAAX box [7]. Sliding of linker regions L1 and L12 in lamin A/C filaments allows linear shortening of the rod domains and confers flexibility to the molecule and elasticity to the whole nucleoskeleton [29]. Desmin forms parallel homodimers that anneal into anti-parallel tetramers by overlapping coil 1B [31]. A total of eight tetramers associate in the so-called unit length filament (ULF), which is the building block of the intermediate filament, formed through longitudinal elongation followed by lateral compaction yielding 10 nm thick filaments [31].

## 3. Striated Muscle Disorders Caused by Mutations in Intermediate Filament Proteins

The interplay between IFs in muscle cells is not limited to a physical interaction aimed at maintaining cell structure and organelle positioning, but also impacts on cellular signaling and gene expression in response to various stress stimuli, cell movement and differentiation [3,15]. A type lamins and desmin share at least two fundamental functions in muscle cells. First, they are both involved in mechanosignaling through interaction with important protein complexes as costameres (at the sarcolemma) or the LINC complex (at the nuclear membrane) [3,25]. Further, A type lamins and desmin contribute to nuclear positioning, a very important function in muscle cells as it specifies myonuclear domains, i.e., the portion of a muscle fiber that is governed by each nucleus [25,32]. As a consequence, altered myonuclear positioning characterizes both laminopathic muscles [25] and desmin null muscle fibers [32]. Further, in muscle diseases caused by mutations in *LMNA*, signaling effectors are aberrantly regulated [33,34,35,36,37,38], while, though less documented, mutations in desmin are expected to disrupt the muscle response to mechanical strain [3,39]. Mutations in *DES* (OMIM *125660) or *LMNA* gene (OMIM *150330) cause striated muscle disorders, including desmin-related myopathy and cardiomyopathy (desminopathy) [40], Emery–Dreifuss Muscular Dystrophy (EDMD2, OMIM # 181350 and EDMD3, OMIM # 616516, due to *LMNA* mutations), *LMNA*-related congenital muscular dystrophy (L-CMD, OMIM # 613205)*,* limb-girdle muscular dystrophy type 1B (LGMD1B) and *LMNA*-linked dilated cardiomyopathy with conduction system defects (CMD1A, OMIM # 115200) [41,42,43,44,45,46]. Recently, synemin mutations (*SYNM* gene, OMIM *606087) have been linked to dilated cardiomyopathy (DCM), while skeletal muscle disease has never been described [11,47]. In particular, four autosomal dominant mutations in *SYNM* have been associated to DCM [48]. Hence, this review will focus on clinical and molecular aspects of skeletal and cardiac muscle diseases due to mutations in *DES* or *LMNA* genes and will suggest pathogenetic hypotheses. Table 1 shows features of skeletal and cardiac muscle diseases caused by *LMNA* and *DES* gene mutations.

## 4. Synemin-Related Cardiac Disorders

**Figure 3:**
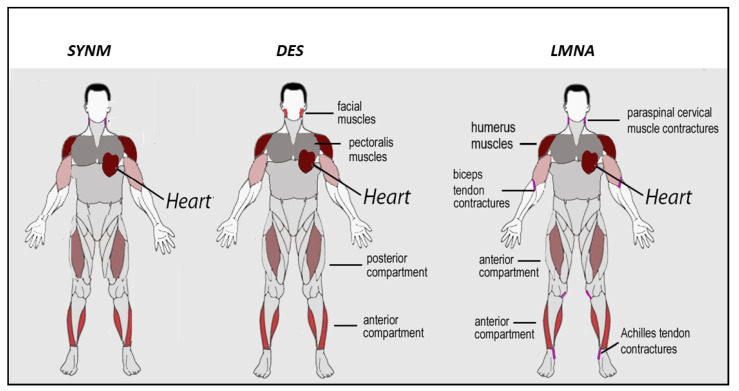
Muscle districts affected in striated muscle disorders caused by mutations in intermediate filament proteins. In the upper row, gene names causative of each group of neuromuscular disorders are indicated (*SYNM*, *DES*, *LMNA*). In the case of *LMNA*-related disorders, sites affected by contractures are also indicated. Achilles tendon contractures may occur, but are infrequent in desminopathy, thus they have not been indicated in the picture.

Mutations in synemin have been associated to dilated cardiomyopathy (Figure 3). In particular, two missense mutations in the protein C-terminus, a frame-shift and a non-sense mutation appear to interfere with protein binding to costameric proteins or protein assembly, respectively [47]. Moreover, *SYNM* has been proposed as a candidate gene for a rare heart-hand syndrome (HHS) referred to as ulnar-mammary-like syndrome [49]. Interestingly, HHS of the Slovenian type has been associated to *LMNA* gene mutations causing production of a short lamin A isoform. HHS typically features cardiac involvement with ventricular tachyarrhythmias, septum fibrosis and dilated cardiomyopathy associated.

## 5. Desminopathies

Desminopathy is a term referred to a skeletal and/or cardiac muscle disorder caused by autosomal dominant mutations in the *DES* gene [3]. Desminopathy is among the myofibrillar myopathies (MFMs), which are hereditary muscle disorders characterized by specific histopathological features of protein aggregation; in particular, focal disintegration of myofibrils predominantly at the Z-disc level and sarcoplasmic protein aggregates that show an accumulation of several proteins, among which is desmin [3,50].

### 5.1. Clinical Aspects of Skeletal Muscle Involvement in Desminopathies

The main muscle districts affected in desminopathy are represented in Figure 3. However, quite variable phenotypes have been associated with *DES* mutations. Autosomal dominant mutations in *DES* were the second cause of MFMs in a large cohort of Spanish patients and the first cause in two different cohorts diagnosed at the Mayo Clinic and Institute of Myology of Pitie’ Salpetriere Hospital, respectively [51,52,53]. Desminopathy usually presents in the third–fourth decade of life, significantly earlier than MFMs due to mutations in *ZASP* and *MYOT* genes characterized by onset in the sixth decade [40,51,54]. Heart involvement is frequently associated, being detected in around 40–90% of the desminopathy cases, according to different studies [40,51,52,54]. Muscle weakness is usually slowly progressive and predominant in distal rather than in proximal muscles and in lower than in upper limbs, at least at the first disease stages; relevant proximal muscle weakness may be observed at later stages [51,53]. Different degrees of weakness are observed among patients, even within members of the same family [40]. In addition, desminopathy can also present with generalized, proximal or scapuloperoneal pattern of weakness. Facial and axial muscles are involved quite frequently [40,51]. Dysphagia is reported in around 40% of the patients [51]. Muscle weakness usually progresses over the years, leading to loss of independent walking ability in around half of the patients [40,51]. Around 15–50% of patients suffer from restrictive respiratory insufficiency [51,53].

Furthermore, desmin was identified as a major protein constituent of inclusions found in muscle fibers of patients previously considered as LGMD1D/1E and autosomal dominant *DES* gene mutations have been linked to LGMD1E [55]. However, LGMD1E and LGMD2R, the autosomal recessive form, have been deleted from the updated classification of LGMDs, being mainly a distal myopathy [56].

### 5.2. Diagnostic Protocols for Skeletal Muscle in Desminopathies

**Figure 4:**
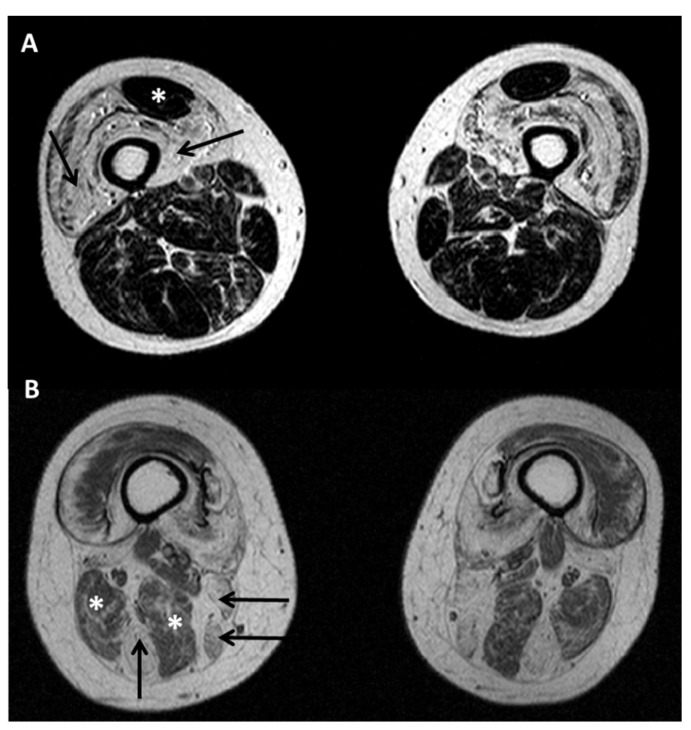
T1 weighted muscle MRI at thigh level in *LMNA* (**A**) and *DES* (**B**) mutated patients. (**A**) Marked fatty infiltration of the vasti (arrows) with sparing of the rectus femoris (asterisk) and of posterior compartment. (**B**) Marked fatty changes in semitendinosus, sartorius and gracilis (arrows) with relative sparing of long head of biceps femoris and semimembranosus (asterisks).

Creatine kinase level is usually normal to moderately elevated in desminopathy. EMG shows a myopathic pattern with spontaneous activity at rest manifested by positive sharp waves, fibrillation potentials, and high bizarre discharges in most cases. Peripheral nerve conduction studies are usually normal. On muscle imaging, peroneal muscles are the first affected followed by involvement of tibialis anterior, while gastrocnemius and soleus muscles were involved in more severely affected patients [57]. At the thigh level, the semitendinosus is at least equally affected as the biceps femoris, followed by sartorius and gracilis, whereas semimembranosus and biceps femoris are relatively preserved for a long time. In the pelvis, earliest changes occurred in the gluteus maximus [57]. Samples of muscle MRI pattern at thigh level in patients with *DES* and *LMNA* mutations are shown in Figure 4. Histological analysis of skeletal muscle tissue is necessary for desminopathy diagnosis, followed by genetic characterization. Desminopathy shows typical histological features of MFMs, including autophagic vacuoles, ectopic expression of multiple proteins in the abnormal fiber regions and myofibrillar disorganization beginning at the Z-disk. Abundant accumulation of desmin-immunoreactive deposits and granulofilamentous material at the ultrastructural level are considered morphological hallmarks of desminopathy [58]. Desmin aggregates contain several proteins, from extracellular matrix components as laminins and collagen VI, to chaperones and linkers as alphaB-crystallin, hsp27 and plectin, up to actin-binding proteins, as actinin and myotilin, and other IFs as synemin, syncoilin and nestin [3].

### 5.3. Clinical Aspects of Cardiac Involvement in Desminopathies

Heart involvement is very frequent in patients with desminopathy, often in combination with the skeletal muscle weakness [40] and impacting significantly on the disease outcome. As for *LMNA*-RD, close cardiac monitoring in desminopathy patients is highly recommended from adolescence [59]. Heart disease in desminopathy may precede, follow or be concomitant with skeletal myopathy, as also observed in *LMNA*-RD, and there is no correlation with the severity of skeletal muscle weakness [51,54,59,60]. In addition, as for laminopathies, cardiac disease in desminopathy may manifest with arrhythmias and/or cardiomyopathy, although arrhythmias are quite rare in desminopathy and frequent in muscular laminopathies [54,59]. Thus, pacemakers are much more frequently implanted than implantable cardioverter-defibrillator (ICD) in desminopathic patients. Cardiomyopathy is usually dilated, but can be sometimes hypertrophic (HCM) or restrictive (RCM) in desminopathy [61], while these forms are very rare in cardiac laminopathies [46,54,62]. In addition, desmin mutations have recently been found in a subset of patients suffering from arrhythmogenic right ventricular cardiomyopathy (ARVC) [63]. Lastly, *DES* mutations have been reported to be a rare cause of left-ventricular non-compaction cardiomyopathy (LVNC), sometimes in association with skeletal muscle involvement [20,64,65]. In a study on the prevalence of desmin mutations in DCM, desmin mutations accounted for up to 2% of disease manifestations [66]. A meta-analysis of 159 patients with 40 different *DES* mutations reported in the literature [54] indicated that up to 50% of carriers had cardiomyopathy, mostly DCM (17%), RCM (12%), and HCM (6%) and to lesser extend ARVC (1%). About 60% of the patients had cardiac conduction disease or arrhythmias, with atrioventricular block as an important hallmark. In a study on 28 patients with a longitudinal follow-up of 10 years, cardiac conduction defect was detected in 25 out of 28, requiring permanent pacing in 16 out of 28 of patients [59]. Notably, among patients presenting at baseline with first degree atrioventricular block, fascicular block, or both, around 40% developed a higher degree of blocking after a mean follow-up of 6.0 years [59]. A recent report of the largest family of a single *DES* mutation (*DES* E401D) causing predominantly inherited ARVC suggested that the prevalence of desmin mutations in ARVC may be higher than expected [67]. In fact, a recent study reported a single L115 *DES* mutation as the cause of ACM in three patients [68]. Interestingly, a patient carrying single heterozygous missense mutations both in *LMNA* and *DES* genes presented at 14 years of age with a rapidly progressive HCM [69]. Desmin accumulation was detected in the skeletal and particularly in the cardiac muscle. The unusual and severe clinical phenotype of this patient was considered as the result of mutations encoding for interacting proteins [69].

### 5.4. Genotype–Phenotype Correlation in Desminopathies

Most of desminopathy cases are dominant de novo in-frame mutations in *DES* gene on chromosome 2q35 [50,70]. A meta-analysis focused on phenotype–genotype correlation revealed that the coexistence of skeletal and cardiac involvement was associated with mutations in coil 2 domain of desmin in around 90% of the cases [50]. Conversely, isolated cardiological phenotype was more frequently found in cases with a mutation in the head or the tail domain [54]. Interestingly, recessive cases of desminopathy have been described in the literature, often with peculiar clinical or histological features. The first recessive desmin-null mutation was reported in two siblings displaying a progressive myopathy associated with muscle fatigue, dysphagia and respiratory involvement [71]. Notably, histological findings showed myopathic abnormalities and cytochrome c oxidase deficient fibers but no MFM pathology. Desmin expression was completely absent on immunostaining and Western blot [71]. Similarly, two cases of autosomal recessive desminopathy with limb-girdle phenotype did not show histological features suggestive of MFMs [72]. Two further brothers with a homozygous deletion of *DES* gene had an EDMD2 phenotype presenting in childhood, including contractures and DCM, without any MFM pathology at muscle biopsy [71]. In addition, two cousins carrying a homozygous truncating *DES* mutation showed clinical and neurophysiological features consistent with neuromuscular junction impairment; one patient improved when administered symptomatic treatment with salbutamol, as in congenital myasthenic syndromes [73]. Similarly, in the recessive desminopathy mouse model with low expression of the mutant R349P desmin protein, the same authors demonstrated neuromuscular endplate pathology [70]. These findings suggested that absence or markedly decreased expression of mutant desmin cause impairment of neuromuscular junction structure and function with clinical picture resembling a congenital myasthenic syndrome [73]. As *DES*, other genes (*BIN1*, *RYR1*, *SCN4A*) usually related to dominant neuromuscular diseases, may cause neuromuscular transmission disorders in recessive cases. Moreover, functional defects and altered nuclear positioning have been described at neuromuscular junctions in EDMD2 muscle [25,33,74].

### 5.5. Pathogenesis of Desminopathies

The prominent feature of desminopathic muscle is formation of desmin aggregates in the cytoplasm, which are caused by assembly defects [75]. These defects are linked to mutations in the coiled-coil domain, which occur in most cases of desminopathy [75]. Desmin aggregates contain several proteins, as listed in paragraph 5.2. Intriguingly, a key role in aggregate formation has been proposed for BAG3 cochaperone, a protein that senses the mechanical unfolding of the actin-crosslinking protein filamin and targets damaged filamin to lysosomes for degradation in the context of chaperone-assisted selective autophagy (CASA), a tension-induced autophagy pathway essential for mechanosignaling in muscle [76]. Of note, mutations in *BAG3* gene have been associated with early onset severe MFM with aggregate formation [77] and even mutations in *CRYAB* gene, encoding for the chaperone alphaB-crystallin, are causative of MFM, pointing to a major role of chaperone proteins in muscular dystrophy and cardiomyopathy pathogenetic pathways (for an updated review see [78]). On the other hand, desmin mutations occurring in the tail domain do not cause abnormal filament aggregation [31], but have been shown to affect the nanomechanichs of desmin network and in particular the response to mechanical strain [79]. Thus, it is conceivable that the ultimate pathogenetic effect of any desmin pathogenetic mutation is the impairment of mechanosignaling [80], which in turn may affect several organelle positioning, including nuclei and mitochondria. In fact, *DES* mutations do not only lead to structural desmin network defects, but also affect mitochondrial positioning, structure and function, which may then generate oxidative stress and cell death [81]. Consistent with a major pathogenetic role of mitochondrial dysfunction, overexpression of Bcl-2 corrects mitochondrial defects and ameliorates inherited desmin null cardiomyopathy [82]. Moreover, MnSOD and/or catalase overexpression in desmin null mouse heart was sufficient to reduce intracellular ROS and improve cardiac function [81]. Formation of desmin aggregates has been also tested in the presence of anti-oxidants, as N-acetyl-cysteine (NAC), in cultured desminopathic cells [60]. Although NAC prevented aggregate formation in desmin mutant cultured cells, it failed to reduce aggregates in mutant muscle [60]. We could hypothesize that rescue of a functional connection between desmin and mitochondria is necessary to avoid accumulation of mitochondrial defects and the oxidative stress condition induced by mutated desmin. Consistent with this interpretation, heart dysfunction that characterizes desminopathic mice was significantly ameliorated by overexpression of alphaB-crystallin, the chaperone protein that binds and co-operates with the desmin network for efficient association and targeting to mitochondria and sarcoplasmic reticulum, required for proper mitochondrial homeostasis [83]. Thus, mitochondrial defects caused by absence of a functional desmin network are a key pathogenetic factor in desminopathy (reviewed in [3,84,85,86]). Of note, as outlined below, overexpression of alphaB-crystallin in laminopathic mice featuring cardiomyopathy obtained amelioration of the cardiac phenotype, showing that even mutated lamins elicit mitochondrial dysfunction dependent on an altered desmin network [87]. Importantly, a rescue mechanism in desminopathic muscle appears to be autophagy, which is induced by accumulation of toxic protein aggregates [88]. Combined treatments with mTOR inhibitors and anti-oxidant molecules have been attempted to reduce the amount of desmin aggregates in *DES*-mutated muscle cells [89]. Importantly, cardiomyocyte death due to the mitochondrial defects caused by the absence of desmin triggers an inflammatory condition involving complement activation and macrophage infiltration in desminopathy preclinical models. [90,91,92]. In particular, overexpression of osteopontin and activation of members of the complement pathway have been demonstrated in desmin deficient muscle [90,91], while overexpression of interleukin 1beta had been previously reported in MFMs [93]. As a whole, the available data suggest that altered mitochondrial function and protein toxicity co-operate with defects in cell nanomechanics and the inflammatory condition to desminopathy pathogenesis.

All the above-mentioned studies of desminopathy pathogenesis have been possible thanks to availability of the mouse *DES* knockout model that faithfully recapitulates desmin-related cardiomyopathy phenotypes [91]. Cardiac-specific overexpression of alpha-B crystallin in this mouse model provided a clear demonstration of the involvement of mitochondrial disfunction in desminopathy and of the role of the desmin chaperone [83]. Moreover, recent advances in the understanding of mutation-specific desminopathy pathomechanisms have been obtained by exploiting induced pluripotent stem cell (IPSCs)-derived cardiomyocytes [20,61,94]. In IPSCs, pathogenicity of the A337P *DES* mutation in LVNC was demonstrated [20] and IFs assembly defects were linked to homozygous Y122H *DES* mutations in the highly conserved coil 1 of the desmin rod domain, causing autosomal recessive RCM with atrioventricular block [61]. Moreover, desminopathic IPSCs- derived cardiomyocytes showed functional defects in vitro, thus representing a good experimental model for testing potential therapeutic treatments [95].

## 6. Muscular Laminopathies

Lamin A/C plays a relevant role in many cellular processes, hence mutations in *LMNA* gene are associated with a wide range of disease phenotypes, ranging from muscular, cardiac, and metabolic disorders to premature aging syndromes [23,43,45,96]. However, the most frequent diseases associated with *LMNA* mutations are characterized by skeletal and cardiac muscle involvement [43].

### 6.1. Clinical Aspects of Skeletal Muscle Involvement in Laminopathies

Main phenotypes associated with skeletal muscle involvement are: limb-girdle muscular dystrophy type 1B (LGMD1B, OMIM # 150330 159001); autosomal dominant Emery–Dreifuss muscular dystrophy (EDMD2, OMIM # 181350) and a form of congenital muscular dystrophy, (L-CMD, OMIM # 613205) [97]. However, these clinical entities may be caused by the same *LMNA* mutation and even coexist in the context of the same family [45,97,98,99]. Therefore, considering the relevant clinical overlap, these phenotypes should be considered as a continuum in the clinical spectrum of *LMNA*-related dystrophy (*LMNA*-RD). Heart is affected in all the three entities, with similar features, except for younger age at onset in L-CMD and EDMD2 than LGMD1B, which also has later presentation of muscle weakness [45,100]. A study involving mainly patients in pediatric age at the end of the follow-up period, showed that cardiac involvement is significantly more frequent in EDMD2 than L-CMD and LGMD1B [101]. Notably, as in desminopathy, cardiac presentation may precede or follow onset of muscle weakness.

EDMD2 was the first described *LMNA*-linked phenotype [41]. The disease is characterized by the triad of early ankles, elbows and spine contractures, muscle wasting and weakness in a scapulo-humero-peroneal distribution, in particular in early disease stages (Figure 3) [102], and cardiac disorders presenting in adult age with dilated cardiomyopathy and conduction system defects associated with high risk of cardiac sudden death [43,100,103,104,105]. Muscle weakness usually occurs within the beginning of second decade, sometimes preceded by contractures, which may be severe and impact on posture and gait [45]. Other forms of Emery–Dreifuss muscular Dystrophy have been linked to lamin-related genes, mostly encoding for proteins of the nuclear membrane [35,44]. Compared to EDMD2, the X-linked Emery–Dreifuss muscular dystrophy (EDMD1) caused by mutations in EMD gene, encoding for emerin [106], displays muscle weakness with humero-peroneal distribution of weakness, contractures as the presenting symptoms, less common loss of walking ability and lower risk of dilated cardiomyopathy and sustained ventricular tachyarrhythmia [46,105,107]. A similar pattern of fatty infiltration in myopathic patients mutated in the EMD and *LMNA* genes have been described through muscle MRI, although involvement of peroneus muscle pointed to EMD gene mutations [108]. Few cases of X-linked EDMD due to mutations in FHL1 gene have been described, differing from EDMD1 and EDMD2 mainly for the detection of HCM [109].

LGMD1B has been deleted by the new classification of LGMDs [56]. However, this clinical entity is still useful to identify the specific pattern of weakness occurring in some *LMNA*-related myopathies. Indeed, LGMD1B differs from EDMD2 by the distribution of muscle wasting and weakness, being characterized by predominant scapular and pelvic girdle muscle involvement. However, in later stages of the disease, differential diagnosis between EDMD2 and LGMD1B may be challenging due to pelvic muscle weakness developing also in the former. Furthermore, LGMD1B age at onset is later than EDMD2, usually in third or fourth decades. Notably, contractures, which were initially considered absent or late in disease course, have been observed in about two thirds of LGMD1B patients, sometimes in early disease stages, although elbow contractures should be considered suggestive of EDMD2 [43,45,110].

Lastly, a congenital form of *LMNA*-RD, the L-CMD, has been reported in patients presenting symptoms at birth or within the first 2 years of life [111,112]. In particular, two phenotypes have been described: a severe congenital form with minimal or absent motor development and a milder and more frequent myopathy with prominent axial weakness manifesting after normal acquisition of head control, defined as “dropped head syndrome”, usually with preservation of walking ability [42,45,101,111]. Rigid spine and scoliosis are relatively frequent [45]; contractures are almost invariably detected, presenting initially in distal limbs, then developing in proximal joints [45,101,111]. L-CMD patients may even progress to EDMD2 or LGMD1B [45,101,111]. Contrary to LGMD1B and EDMD2, respiratory failure is very frequent in L-CMD, whereas cardiac involvement seems to be less common, but this may be related to the young age of the patients when evaluated. However, sudden death has been reported even in the first decade of life and a study focused on pediatric patients with L-CMD showed dysrhythmias in around half of the cases at a mean age of 14 years [111,113]. Muscle biopsy revealed dystrophic features in more than a half of the patients, with inflammatory findings relatively uncommon [111]. However, *LMNA* mutations were detected in about half of the cases in a small cohort of Japanese patients presenting symptoms before the age of 2 years and suspected to have an inflammatory myopathy according to histological findings at muscle biopsy; some benefit from steroids treatment was observed in four out of the eight treated cases [114]. Similarly, two out of four pediatric L-CMD patients in a Chinese cohort clearly improved with corticosteroid treatment [101]. Central nervous system is not affected in L-CMD, except for a single case report on a girl with dropped head syndrome and focal white matter changes without cognitive impairment [115].

In *LMNA*-RD, creatine kinase is usually normal or mildly elevated (maximum 5 times upper normal value), while higher values can be observed in L-CMD [101,111]. Electromyography is usually not relevant for diagnosis, showing aspecific myopathic patterns, but may be helpful in differential diagnosis with neurogenic diseases. Muscle MRI may be helpful in differential diagnosis towards other myopathies (Figure 4), being *LMNA*-RD associated with predominant fatty infiltration of medial gastrocnemius and vasti muscles with relative sparing of the rectus femoris [116,117]. Notably, muscle MRI pattern at thigh and calf levels is similar in EDMD, LGMD1B and advanced stage L-CMD patients, whereas the involvement of the vasti muscles is not prominent in the early stage of L-CMD [118]. Whole body MRI in pediatric *LMNA*-RD displayed predominant fatty changes in erector spinae, serratus anterior, subscapularis, gluteus medius and minimus, vastii, adductor magnus and longus, semimembranosus, medial gastrocnemius, and soleus muscles [108].

Histological findings in the skeletal muscle tissue of *LMNA*-RD patients are usually unspecific, displaying dystrophic features only in some cases, mainly affected by L-CMD, making muscle biopsy not necessary for diagnosis in patients with typical clinical features [45]. However, clustered nuclei and nuclear dysmorphism can be observed in EDMD2 muscle and focal heterochromatin loss at the nuclear periphery can be detected by electron microscopy analysis [25,119]. Western blot analysis for lamin A/C detection [112] showed reduced protein levels only in around half of the cases; hence, also taking into account the required technical expertise, its role in the diagnosis workup of *LMNA*-RD should not be considered as relevant for diagnostic purposes.

A study investigating a large cohort of Italian *LMNA*-RD patients demonstrated that LGMD1B was by far the most frequent muscle phenotype and confirmed that the natural history of *LMNA*-RD is mainly marked by heart involvement and related complications [45]. Conversely, a recent study investigating a large cohort of Chinese patients with *LMNA*-RD, showed a higher frequency for L-CMD, followed by EDMD2 and LGMD1B [101]. In the Italian cohort of *LMNA*-RD patients, progression of muscle weakness was slow over the years and only 6 out of 75 patients became wheelchair bound after a mean period of about 20 years from disease onset [45]. Apart from L-CMD, respiratory involvement was usually mild and assisted ventilation was required in a minority of patients [45]. The study on Chinese patients showed similar data, with loss of ambulation mostly observed in L-CMD cases who had achieved the ability to walk [101].

Hereditary axonal neuropathy has been reported in a few families carrying the same homozygous *LMNA* mutation (R298C) and originating from north-west Africa, diagnosed as CMT2B1 [120,121,122]. A family originating from the south west of France and mutated in *LMNA* gene showed axonal sensorimotor neuropathy in association with muscular dystrophy, cardiac disease, and leuconychia [123]. In addition, a small number of patients (8%), having a myopathy due to *LMNA* mutation, may show unspecific nerve involvement [45], mainly axonal, although it is still not clear if the neuropathy is coincidental or pathologically related to *LMNA* mutations.

Overlapping syndromes are characterized by the co-occurrence in the same subject of different diseases caused by a pathogenic variant of the lamin A/C gene; metabolic alterations in association to skeletal and/or cardiac alterations proved to be the most frequent overlapping syndromes. For instance, partial lipodystrophy, hepatic steatosis and/or hypertriglyceridemia can be associated with dilated cardiomyopathy or conduction system defects, with or without muscular disease [124]. Other overlapping syndromes may include skeletal and/or cardiac muscle disorders in association with neuropathy and dermatologic abnormalities, or associated with metabolism disturbances and lipodystrophy [28,125,126]. For instance, Mandibuloacral Dysplasia, a syndromic laminopathy characterized by bone alterations, lipodystrophy, metabolic defects and premature ageing, with or without dermatologic abnormalities, variably associates with skeletal and cardiac disease [28,125,126].

### 6.2. Clinical Aspects of Cardiac Muscle Involvement in Laminopathies

Mutations in the *LMNA* gene very often affect the cardiac and/or cardiovascular system [23,35,45,46]. In laminopathies, heart involvement can be isolated or associated to disorders in other tissues, mainly in combination with myopathy [97]. Heart disturbances occurring in *LMNA*-RD are due to structural and functional abnormalities of the electrical and mechanical systems, mainly causing dilated cardiomyopathy and arrhythmias [46,100]. Cardiac disorders show a high penetrance and almost all *LMNA*-RD patients after the age of 30 years show heart disease, mainly arrhythmias or ACM, regardless the presence of a myopathy [100,127]. As for desminopathy, cardiologic follow-up is strongly recommended in *LMNA*-RD patients, even in pediatric age. However, in most *LMNA*-RD cases with muscular dystrophy, cardiac phenotype presents around ten years after the presentation of skeletal muscle manifestations [100]. Family history for cardiac disease is often reported [100]. Usually, the first signs of cardiac involvement are electrical abnormalities, which can include low P wave and prolonged PR interval, with a narrow QRS complex [128]. Patients often start showing various rhythm disturbances, which can be unspecific like sinus bradycardia, sick sinus syndrome, bundle branch block, supraventricular and ventricular ectopic beats, progressive atrioventricular block [129]; although rarely, patients may also develop an atrial paralysis, a severe modality of cardiac compromise typical for cardiolaminopathies [127]. Furthermore, mutations in *LMNA* represent around 2–5% of LVNC cases [104,130]. Several studies showed how cardiac compromise seem to be related with the age with only a small percentage of patients developing cardiac rhythm abnormalities within the first decade and the majority of patients showing electrical alterations after the third decade [127]. In addition, the coexistence of *LMNA* mutations and congenital heart defects or aortic involvement have been recently reported in pediatric patients [131]. Later on, the modality of cardiac compromise of affected subjects may be further complicated by cardiomyopathy leading to heart failure, usually by middle age [96,132]; this condition, which occurs less frequently than cardiac rhythm disturbances, is usually preceded by dysrhythmias and atrial tachiarrhythmias [46]. Mutations in the *LMNA* gene are detected in around 5% of patients affected by non-familial dilated cardiomyopathy, 5–10% of patients with familial dilated cardiomyopathy and 33% of cases of familial dilated cardiomyopathy with conduction system defects [104,132]. Several studies aimed at reporting the natural history of cardiolaminopathies have shown how patients may die suddenly, even despite the implantation of a pacemaker, making ICD the gold-standard to prevent sudden death in these patients [100,104]. This may be explained by the relatively frequent rate of malignant ventricular tachyarrhythmias, even in the absence of dilated cardiomyopathy [104]. It is not possible to predict the extent and severity of cardiac compromise solely on the basis of the characteristic of the *LMNA* gene mutation, as confirmed by the marked phenotypic variability among subjects bearing the same lamin A/C gene mutation [46]. Life style and other variables may modulate the deleterious effect of the mutation, thus helping the clinician in the care of cardiolaminopathies; in this regard, it has been shown that a previous history of competitive sport can produce a deleterious effect in subjects carrying a lamin A/C mutation [133]. Furthermore, sustained malignant ventricular arrhythmias tend to occur in subjects bearing at least two of the following variables: the status of carrier of an *LMNA* gene mutation other than missense, the male gender, a previous history of non-sustained ventricular arrhythmia and an ejection fraction less than 45% at the first cardiac detection [134]. Hence, the presence of at least two of the previous risk factors strongly support ICD implantation, as recommended by the international guidelines [104]. A recent study on a large cohort of *LMNA*-RD patients revealed that even tendon retractions at baseline evaluation show a significant association with major cardiac events, such as heart transplantation, malignant ventricular arrhythmias and cardiac death [100]. Conversely, the aforementioned study did not confirm the gender effect and showed only a poor predictive value of mutation type in *LMNA*-related cardiomyopathy risk stratification [100].

### 6.3. Genotype–Phenotype Correlation in Muscular Laminopathies

Mutations in the *LMNA* gene associated with striated muscle involvement are mostly autosomal dominant. The vast majority are in-frame missense mutations, which can occur all along the *LMNA* gene. However, a few muscular dystrophy cases caused by recessive *LMNA* mutations have been described and defined as EDMD3 [43]. These cases usually displayed severe contractures, whereas cardiac involvement, if any, was only late and mild.

The relevant clinical variability associated with LMNA mutations, also observed within the same family, makes it challenging to establish genotype–phenotype correlations in *LMNA*-RD [135]. In an Italian retrospective study of a cohort of 78 *LMNA*-RD patients, *LMNA* missense mutations were mainly identified in patients with EDMD2, in those affected by L-CMD and in patients without cardiac involvement, as already suggested by a previous study, whereas frameshift mutations were more frequently detected in LGMD1B patients and in those with heart involvement [45]. In EDMD2 and LGMD1B patients, gene variants mainly clustered in the immunoglobulin-like (exon 7-10) and coil 2B (exon 6) regions, respectively, which are regions crucial for the interactions of lamins with the inner nuclear membrane proteins [45]. Mutations associated with L-CMD were spread across the N-terminal and the first part of the rod domains (exon 1 and exons 4 and 5), which are involved in dimer formation. These mutations affect the overall organization of the nuclear envelope and in nuclei of cultured fibroblasts derived from patient skin biopsies giving rise to the so-called honeycomb structures detectable by lamin A/C or emerin antibodies [24]. Mutations restricted to the tail domain were not significantly associated with heart involvement, suggesting that myocardium might be more sensitive to modifications in the N-terminal portion of lamin A/C than skeletal muscle [136]. Non-sense and out-of-frame mutations are extremely rare in muscular laminopathies and detected almost exclusively in patients with skeletal muscle involvement [137].

Environmental factors and possible genetic modifiers have been postulated to explain the phenotypic variability. In particular, some studies revealed a possible pathogenetic role for genes that can modulate the effect of lamin A/C mutations or a digenic mechanisms, by which mutations in *LMNA* and in another gene may coexist in the same patient [69,138,139]. In this respect, double pathogenetic mutations have been reported in lamin A/C and nuclear membrane proteins as emerin, SUN1, SUN2 or nesprins [26,69] or cytoskeleton components including desmin [69] and titin [139], which may provide further clues to explain the phenotypic variability of muscular laminopathies [26]. A recent study based on a primer library designed on the basis of functional lamin interactions and candidate sequences emerged from EDMD2 RNA sequencing studies has revealed that genes from the extracellular matrix to the nuclear envelope are directly or indirectly involved in EDMD2 pathogenesis [140].

### 6.4. Pathogenesis of Muscular Laminopathies

The pathogenesis of muscle laminopathies has been investigated from several points of view. Early studies have implied lamin A/C in chromatin organization and altered heterochromatin arrangement has been shown in EDMD2 [24]. Thus, not surprisingly, altered gene expression profiles have been reported in preclinical models of muscle laminopathies [140,141]. In EDMD2, muscle-specific gene repositioning, which occurs upon differentiation stimuli and involves lamin A/C-chromatin interactions, is affected [140]. This condition is not only due to the presence of mutated lamin A/C, but also to co-occurrence of variants in genes encoding for proteins of the nuclear envelope, as recently reported in EDMD2 and EDMD2-like patients [140]. Moreover, dysregulation of epigenetic enzymes as the Polycomb group transcriptional repressors and the acetyltransferase HDAC2 has been implicated in aberrant gene expression in EDMD2 models [142,143,144]. Among affected genes, also desmin has been identified, and altered desmin expression has been shown to impair myogenic differentiation of laminopathic myoblasts [141]. However, the main involvement of the IFs network in EDMD2 pathogenesis appears to occur in the frame of the complex and finely regulated mechanosignaling system that transmits mechanical forces from outside the muscle cells to the nucleus to link gene transcription to contraction. In this context, two major categories of molecules come into play, the LINC complex, formed by nuclear transmembrane proteins linking lamins to the cytoskeleton [25,26], and the PI3-K and MAPK signaling effectors, which are not only affected in laminopathic muscle, but are also therapeutic targets [37] currently tested in clinical trials (https://www.clinicaltrials.gov/ct2/show/study/NCT03439514, accessed on 16 April 2021). It is noteworthy that the LINC complex, a platform of nuclear envelope transmembre proteins connecting the nucleoskeleton to the cytoskeleton, encompasses the nesprins, which bind desmin in skeletal (nesprin 1/2) and cardiac muscle (nesprin 3) [6,8]. Binding is mediated by the giant linker protein plectin and involves a complex interaction with microtubules, which is needed to keep nuclear morphology, chromatin organization and gene expression in cardiomyocytes [8]. Importantly, loss of this equilibrium due to either desmin depletion or nesprin 3 downregulation may cause DNA damage [8]. Thus, desmin and nuclear envelope proteins co-operate in maintenance of nuclear shape and chromatin functional organization in cardiac myocytes.

The pathogenesis of muscular laminopathies is further driven by systemic factors that affect muscle turnover and cause profibrotic processes. Among altered circulating molecules are transforming growth factor beta 2 (TGFbeta 2), interleukin 17 (IL-17) and granulocyte colony-stimulating factor (G-CSF) [145,146]. Downstream of TGFbeta 2 activation, fibrotic processes are triggered in skeletal and cardiac muscle cells, and can be attenuated by either inhibition of TGFbeta 2 [145] or blockade of the connetive tissue growth factor (CTGF), which is activated through TGFbeta 2 signaling [147]. In a large cohort of laminopathic patients presenting with skeletal and/or cardiac muscle involvement, cytokine profiles were able to discriminate different disease patterns. In particular, IL-17, G-CSF and TGF-β2 levels differed significantly between *LMNA*-RD patients and controls, whereas interleukin-1β (IL-1β), interleukin-4 (IL-4) and interleukin-8 (IL-8) were differentially expressed in *LMNA*-RD patients compared to those affected by laminopathies without muscle involvement [146]. IL-17 was significantly higher in LMNA-RD patients with associated cardiac disease [146]. In addition, the up-regulation of Toll-like receptors (TLRs) 7 and 9, which are mediators of innate immunity, in the muscle of patients with *LMNA*-RD compared to other myopathies, further supports a role of inflammation and immunity in the pathogenesis of *LMNA*-RD [148].

Several preclinical models of EDMD2, some of which are mentioned below, have provided further important insights into disease pathomechanisms. As for many other cardiac diseases, induced pluripotent stem cells (IPSCs) differentiated into cardiomyocytes have been particularly useful for unraveling mechanisms related to the onset of *LMNA*-linked cardiomyopathy [94]. In laminopathic IPSCs-derived cardiomyocytes carrying the K219T-*LMNA* mutation, altered action potential, reduced peak sodium current and diminished conduction velocity have been shown and linked to epigenetic downregulation of Nav1.5 sodium channel expression due to increased binding of Lamin A/C to the promoter of SCN5A gene [149]. Interestingly, defects in SCN5A had been associated with ventricular arrythmias in a patient carrying the V445E LMNA mutation and showing LVNC cardiomyopathy [130]. An important study has recently shown loss of cell identity in *LMNA*-mutated IPSCs-derived cardiomyocytes, supporting the above-mentioned involvement of lamin A/C in tissue-specific gene regulation and impairment in muscular laminopathies [150]. Animal models of *LMNA*-related cardiomyopathy have been established either by knockin of orthologous human pathogenetic mutations or by partial or complete ablation of *Lmna* gene [142,151]. The *Lmna* ^H222P/H222P^ mouse model of EDMD2, which features dilated cardiomyopathy and shows different phenotypes in the homozygous and in the heterozygous state, has been extensively characterized [151]. ERK 1/2- MAPK signaling defects, TGFbeta-dependent activation of profibrotic processes, defects in cytoskeleton dynamics involving the actin-depolymerizing protein cofilin-1, defects in the desmin network and mitochondrial dysfunction have been found in *Lmna* ^H222P/H222P^ myocardium, and elevated TGFbeta2 levels have been measured in mouse serum, providing a complex picture of *LMNA*-related cardiomyopathy pathomechanism that links altered signaling regulation to fibrosis and defects in cytoskeleton dynamics, leading to mitochondrial defects and oxidative stress [87,145,147,151,152]. Those studies have also suggested a number of potential therapeutic strategies [145,147,151]. In *Lmna* delta8-11^-/-^ mice, which also feature cardiomyopathy and muscular disease and have been characterized for several phenotypic aspects, a link has been recently demonstrated between loss of lamin A/C and activation of aberrant gene expression pathways ultimately leading to muscle stem cell depletion [144].

## 7. Pathogenetic Hypotheses Based on Desmin-Lamin A/C Interplay

Several findings obtained in laminopathic or desminopathic muscle suggest that a functional interplay of desmin and lamin A/C disrupted under pathological conditions may contribute to the pathogenesis of both muscular laminopathies and desminopathy. This hypothesis is strongly supported by the occurrence of severe forms of muscular dystrophy due to *LMNA* and *DES* mutations [69].

**Figure 5:**
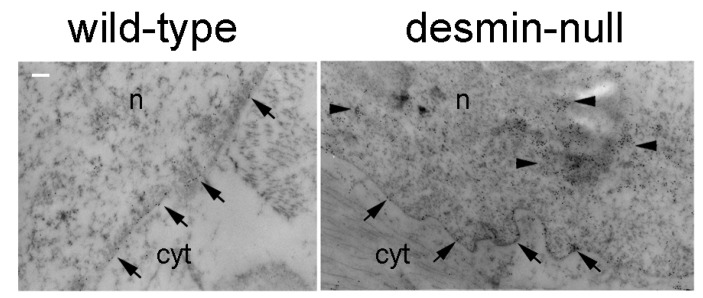
Nucleoplasmic localization of lamin A/C is increased in the nuclei of desmin deficient cardiomyocytes. Electron microscopic analysis of cardiac tissue sections from wild-type and desmin-null mice, immunogold-labeled for lamin A/C. Lamin A/C-bound gold particles are evident at the nuclear periphery (arrows) of the wild-type nucleus and appear reduced in desmin-null nucleus. Nucleoplasmic accumulation of lamin A/C-bound gold particles is observed in the desmin-null nucleus (arrowheads). (n, nucleus; cyt, cytoplasm). Scale bar, 100 nm.

*Lmna* null mouse cardiomyocytes show detachment of desmin IFs from the nuclear surface and disruption of the desmin network [153]. Formation of desmin aggregates has been also observed in laminopathic muscle [87]. These data suggest that the complex network formed by desmin and its partner proteins in muscle cells requires a functional lamina as part of a unique intracellular structure. On the other hand, desmin appears to influence lamin A/C intranuclear. In Figure 5, we show lamin A/C localization in *DES* null cardiomyocyte nuclei. While in wild-type mouse nuclei, the vast majority of lamin-bound gold particles decorate the nuclear periphery, nucleoplasmic distribution of lamin A/C is clearly observed in desminopathic nuclei (Figure 5). This observation raises the inttriguing hypothesis that modulation of desmin levels (and/or interactions) might contribute to nuclear lamina remodeling. Along this line, both lamin A/C and desmin have been linked to nuclear morphology and chromatin structure and function [8,154], and both lamin A/C and, unexpectedly, desmin have been involved in transcriptional regulation [8,16,140,141]. Loss of lamin A/C or pathogenetic *LMNA* mutations impair skeletal myoblast differentiation due to transcriptional impairment [140,141]. Similarly, inhibition of desmin expression in C2C12 cells blocks myogenic differentiation and interferes with the myogenic transcriptional regulators myoD and myogenin [155]. In addition, desmin deficiency blocks the myogenic [156] and cardiogenic [16,84,157] pathways during embryonic stem cell differentiation. Of note, desmin is among downregulated genes, in *Lmna* null myoblast, and myogenic differentiation can be rescued by desmin overexpression [141]. In a recent study performed in *Lmna* ^H222P/H222P^ mice, which carry an EDMD2 mutation and feature cardiomyopathy and muscular dystrophy, desmin toxicity due to formation of cytoplasmic aggregates was clearly shown [87]. In fact, genetic downreguation of desmin levels or rescue of normal desmin network by overexpression of the desmin chaperone alphaB-crystallin, ameliorated most structural defects and the cardiac phenotype [87]. Thus, even in EDMD2, a crucial determinant of the pathogenetic pathway involving desmin are the mitochondrial defects emerging from the destabilisation of the desmin network caused by the mutant lamin A/C. AlphaB-crystallin has the ability to correct such defects [83] protecting the cardiac tissue [87].

On the other hand, in desmin deficient muscle, some pathogenetic mechanisms common to those observed in laminopathic muscle have been reported. Importantly, nuclear positioning was altered both in desminopathic and laminopathic muscle cells, as shown in desmin null cardiac and skeletal muscle [32,154] and EDMD2 striated muscle [25,139,158]. In EDMD2 muscle cells, myonuclear clustering was observed and linked to altered interplay of lamin A/C and prelamin A with the nuclear envelope protein Samp1 [158] and the LINC complex proteins SUN1, nesprin 1 and 2 and SUN2 [25]. Similarly, the association of desmin with lamins through the LINC complex, and in particular by nesprins binding, supports a proper coupling between the cytoplasm and nuclear interior. Its absence may lead to the altered nuclear positioning observed in desmin deficient muscle. In support of this hypothesis, it has been shown that desmin knockdown in cultured cardiomyocytes alters nuclear morphology and chromatin organization, even affecting LADs and this effect is phenocopied by nesprin 3 knockdown [8].

Moreover, cardiomyocytes bearing either desmin or lamin A/C mutations show altered intercalated disk structure [22,95]. Although defects in intercalated disks have been linked to reduced connexin 43 levels and altered alpha-tubulin interplay in laminopathic heart [22], a role for desmin in defects of intercalated disks occurring in EDMD2 preclinical models cannot be ruled out and desmin interactions at the desmosomes of EDMD2 cardiomyocytes warrant investigation.

Further hints in favour of common pathogenetic mechanisms of muscular laminopathies and desminopathy involve the activation of inflammatory and profibrotic pathways partially or exclusively triggered by the defective myocytes. In fact, both muscular laminopathies and desminopathies have defects in the secretome, with activation of an inflammatory and profibrotic response involving TGFbeta 2 and other cytokine increase, as in *LMNA*-RD [145], or activation of the complement and osteopontin overexpression in the myocardium of mice featuring desmin-related cardiomyopathy [90,91]. In desminopathies and desmin-null mice, it has been shown that cardiomyocyte death activates an inflammatory pathway and causes secretion of pro-inflammatory molecules. However, we cannot rule out a transcriptional effect, at least of mutated lamin A/C, on the expression of circulating factors as TGFbeta 2 and other cytokines [145,146,148,159]. The pathogenetic relevance of those pro-inflammatory conditions has been shown by inhibiting TGFbeta 2 or CTGF in laminopathic muscle cells and mouse models [145,147] or complement components and osteopontin in desminopathic muscle [90,91]. In particular, a TGFbeta 2 neutralizing antibody elicited reduction of the hypersecreted collagen I and alpha- Smooth Muscle Actin, a marker of profibrotic myofibroblasts, in laminopathic skeletal muscle, while CTGF inhibition in laminopathic mouse myocardium by using an anti-CTGF antibody decreased myocardial fibrosis and improved the left ventricular dysfunction [145,147]. On the other hand, in the desmin deficient mice, while overexpressed osteopontin induced secretion of profibrotic galectin-3 by infiltrating macrophages and promoted cardiomyopathy, inhibition of osteopontin or complement components C5a (CD88) and C5ar improved the disease [90,91,92].

In conclusion, the published studies described above strongly suggest that better understanding of the mechanisms leading to cardiomyocyte death due to IF network defects could be very important for the development of the proper therapeutic strategies for genetic and secondary IFs disorders. Thus far, preclinical studies following inhibition of pathways leading to mitochondrial defects are very promising. Similarly, better understanding of potentially involved circulating molecules and related mechanosignaling pathways could be relevant for therapeutic strategies for genetic and secondary IFs disorders, as indicated by the published studies showing beneficial effects of TGFbeta 2 or CTGF inhibition in EDMD2 preclinical models [145,147]. In this respect, although drugs able to counteract IFs-related diseases are not currently available, and a few molecules are being explored in clinical trials, it is worth mentioning that beneficial effect of the inhibition of mTOR pathway with autophagy activation, has been also demonstrated in preclinical models of *DES* or *LMNA*-linked myopathy [35,88,89,160,161], suggesting that detoxification is also relevant to phenotype improvement.

## 8. Therapeutic Perspectives

No causative therapy is available for *LMNA*-RD and desminopathies to date, hence only symptomatic approaches are available, including management of respiratory, cardiac and ortopedic complications. In particular, cardiac disease treatment is based on current clinical practice, also including pacemaker or ICD implantation and heart transplantation in the most severe cases [46,100,104]. Potential treatments for desminopathy have been explored by testing drugs that avoid desmin aggregate formation as antioxidants and stimulators of macroautophagy [89]. Ectopic increase of alphaB-Crystallin levels has been demonstrated to improve the heart phenotype both in desminopathy and in EDMD2 models, suggesting another potential therapeutic strategy [87]. On the other hand, occurrence of fibrotic processes in laminopathic and desminopathic muscle suggests that drugs inhibiting profibrotic signaling could be also useful [145,147]. Among potential molecules, anti-TGFbeta2 neutralizing antibodies and small molecules inhibiting pERK 1/2, Smads and PI-3K signaling have been proposed and some of them are being tested in clinical trials for *LMNA*-related cardiomyopathy (https://www.clinicaltrials.gov/ct2/show/NCT03439514, accessed on 16 April 2021) [145,147]. The use of steroids, expecially in the severe early onset cases of L-CMD with dropped head syndrome, has been also suggested, based on the occurrence of some inflammatory condition in muscles [162]. As secondary inflammatory processes and cytokine secretion may also affect desmin structure and localization at intercalated disks, as shown upon TNF-α-induced caspase cleavage of desmin in heart failure models [84], anti-inflammatory treatments could be also suggested in desminopathy. Inhibition of complement hyperactivation observed in *DES* null mice and *DES*-related ACM is also a promising therapeutic option [90]. A further approach was focused on the neuromuscular junction (NMJ) through the administration of an adeno-associated virus vector encoding the human DOK7 gene, whose expression is essential for the normal formation of the NMJ, to EDMD2 model mouse [4]. This model, showing structurally and functionally impaired NMJs, clearly benefited by DOK7 gene expression, resulting in enlargement of NMJs and positive effects on motor function and life span [163].

## 9. Concluding Remarks

**Figure 6:**
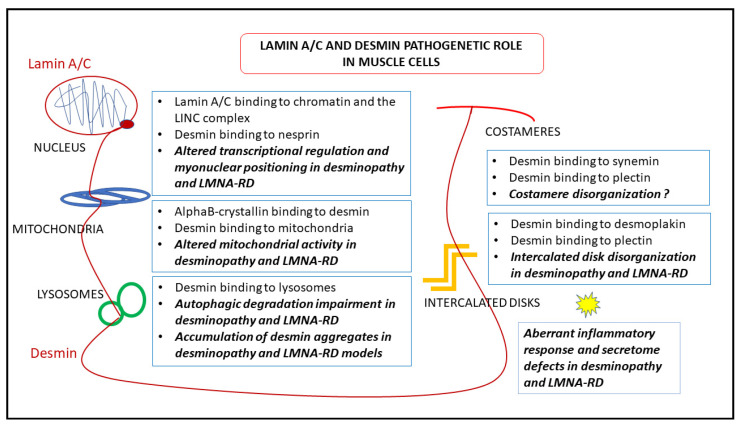
Lamin A/C and desmin potential pathogenetic role in muscle cells. Intracellular structures involved in lamin A/C and/or desmin-related functions and pathogenetic mechanisms related to each function are schematically represented. Lamin A/C and desmin are represented as dark red filaments. Desmin filaments connect all represented organelles and structures in skeletal or cardiac muscle (intercalated disks specifically in the heart). Each box refers to the adjacent structure. Pathogenetic events are written in bold characters in each box. Downstream of all pathogenetic events, aberrant inflammatory response and secretome defects contribute to disease progression (lower right box).

In healthy muscle, desmin and lamin A/C are main components of a dynamic filament network involved in mechanosignaling, nuclear positioning and mitochondrial homeostasis. Thus, both cellular homeostasis and cellular response to external signals rely on a resilient and dynamic intermediate filament structure spanning from the cell membrane up to the nuclear interior. This is made possible by a number of highly specialized lamin and desmin interactors in each cellular compartment, including the LINC complex that binds lamin A/C at the nuclear periphery and desmin in the cytoplasm through the adaptor protein plectin, the synemins that connect together with desmin to the costamers, and desmoplakin at the intercalated disks that joins desmin to the whole junctional system of cardiomyocytes. Further, direct interaction of lamins with nuclear structures as chromatin and the nuclear envelope, and of desmin with contractile apparatus, mitochondria and other membranous organelles and sarcolemma, ensure surveillance of positioning and function of those structures. As a whole, lamin and desmin play major roles in muscle differentiation, maintenance of muscle cell niches, intra- and intercellular communication and response to stress—not only mechanical stress, but even oxidative stress occurring under physiological muscle activity or pathological conditions. In desminopathic and laminopathic muscle, lamin and desmin functionality as well as lamin–desmin interplay are disturbed, causing both structural and functional muscle abnormalities, ultimately eliciting myocyte death, activation of inflammation and the associated secretome alterations (Figure 6).

An intriguing hypothesis has been recently proposed that desmin gain of function leading to aggregate formation coupled with desmin loss of function elicits the most deleterious condition in desminopathic heart as well as in failing heart [2]. Analogously, both mutated lamin gain of function leading for instance to LINC complex engagement and lamin loss of function manifested by impaired transcriptional regulation or nuclear positioning appear to contribute to *LMNA*-RD pathogenesis. These conditions make really hard to identify a proper therapeutic strategy, and suggest that, while searching for gene editing approaches, targeting downstream pathogenetic pathways related to inflammation and secretome abnormalities may be a more realistic therapeutic option.

## Figures and Tables

**Table 1:** Clinical features of *LMNA* or *DES*-related striated muscle diseases.

Feature	*LMNA*	*DES*
Inheritance	AD	AD
Age at onset	at birth to 4th decade	3rd–4th decades
Distal muscle weakness	rare, frequent only in EDMD2	frequent
Cranial nerve involvement	rare	relatively frequent
Contractures	frequent	very rare
Scoliosis/rigid spine	frequent	absent
Loss of independent ambulation	rare and late *	frequent and late
Respiratory insufficiency	rare, mainly in L-CMD	relatively frequent
Heart involvement	very frequent	very frequent
Cardiomyopathy	DCM, ACM, rarely LVNC	DCM, ACM, less frequently RCM, HCM, rarely LVNC
Need of ICD/PM	frequent, mainly ICD	frequent, mainly PM
Serum CK	normal or mildly elevated, high in L-CMD	normal or mildly elevated
Spontaneous activity at EMG	rare	frequent
Skeletal muscle histology	unspecific	MFM changes **
CNS involvement	no	no
Type of predominant mutations	Missense	Missense

AD: autosomal dominant; DCM: dilated cardiomyopathy; ACM: arrhythmogenic cardiomyopathy; RCM: restrictive cardiomyopathy; HCM: hypertrophic cardiomyopathy; LVNC: left-ventricular non-compaction cardiomyopathy (LVNC); ICD: implantable cardioverter-defibrillator; PM: pacemaker; CK: creatine kinase; L-CMD: *LMNA*-related congenital muscular dystrophy; EMG: electromyography; MFM: myofibrillar myopathy; CNS: central nervous system. * L-CMD patients frequently do not achieve independent walking. ** MFM changes include focal disruption of myofibrils mainly at the Z-disc level and sarcoplasmic protein aggregates often with rimmed vacuoles and showing an accumulation of several proteins, including desmin.

## Data Availability

Data sharing is not applicable to this article.
